# Advanced Practitioners’ Favorite Books: The Sequel

**Published:** 2014-03-01

**Authors:** The Editors of JADPRO

Several issues ago, we published a list of advanced practitioners’ favorite books. We received so much positive feedback about that list that we thought we’d do it again. The holidays are over, and the frenetic pace of those hectic months is hopefully lessening. Now might be the time to find a new (or new to you!) book and relax, learn something new, or just be entertained. We’ve selected a few of our favorites and are hoping you’ll find something of interest. Although oncology and health are dominant themes in this list, we’ve sprinkled in a few titles on other topics as well. So make a cup of tea, find a cozy spot, and get reading!

## Salt Sugar Fat: How the Food Giants Hooked Us

**Michael Moss** 

If you were planning a special dinner party, you might want to wait until after your event to start this well-researched book on the processed food industry and its effects on our health and weight. You’ll be entertained yet horrified as the author describes the effects that salt, sugar, and fat have on our well-being. Obesity is a growing epidemic, one that has been linked to the processed food companies. Michael Moss, a Pulitzer Prize–winning journalist for The New York Times, describes how the industry has been scientifically engineering foods to induce our cravings to overeat. This compelling book is a wake-up call as well as a reminder that we are not helpless in our ability to resist.

**Figure 1:**
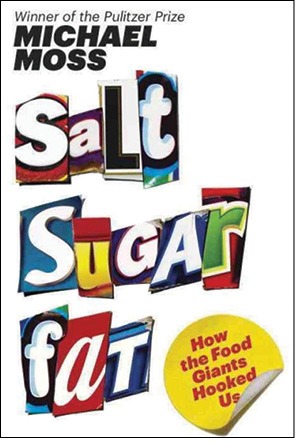
Salt Sugar Fat: How the Food Giants Hooked Us

## The Cancer Chronicles: Unlocking Medicine’s Deepest Mystery

**George Johnson** 

A science journalist for The New York Times, George Johnson has turned his personal experience with cancer into a deft exploration of this often-devastating disease and its treatment. When his wife was diagnosed with metastatic cancer, the author began to research the disease in earnest, trying to understand and learn about this formidable foe. He explores clinical trials and experiments, noting that our knowledge of this dreaded condition has expanded through the advances of cancer research and new therapies. Like Johnson, you might not find all the answers, but you will learn a lot about cancer from the perspectives of both patients and health-care professionals in this engaging yet informative book.

**Figure 2:**
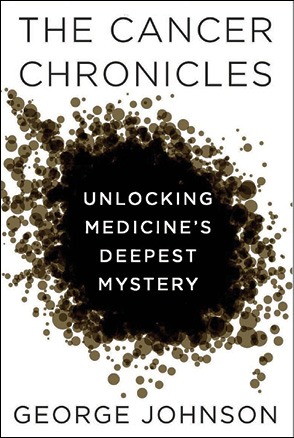
The Cancer Chronicles: Unlocking Medicine’s Deepest Mystery

## Five Days at Memorial

**Sheri Fink** 

This riveting read is a thought-provoking exploration of 5 days at Memorial Medical Center in New Orleans following the devastation of Hurricane Katrina. After the epic storm, Memorial experienced power failure; caregivers for the patients unable to be evacuated were exhausted and at the limits of their endurance. Some of the health-care professionals were accused of injecting specific patients with drugs to help speed up their deaths while trying to deal with the ethical and physical nightmares the storm had produced in their facility. Although readers will probably already have heard about the legal decisions made regarding these workers, the reporting of the events at Memorial is incredibly detailed and fair, leading to a superb book about this unforgettable tragedy.

**Figure 3:**
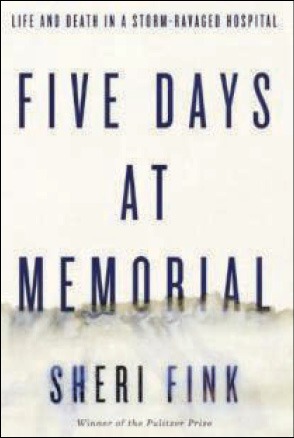
Five Days at Memorial

## The Philadelphia Chromosome: A Mutant Gene and the Quest to Cure Cancer at the Genetic Level

**Jessica Wapner** 

Anyone who took care of patients with chronic myeloid leukemia (CML) prior to the development of imatinib remembers the frustration of a disease that was essentially fatal. In her truly exciting book, Jessica Wapner describes the development of this revolutionary drug, which ushered in the age of molecular targeted therapy. This agent’s success did not come overnight; the author skillfully discusses the science and people responsible for the cure for CML. Although the chromosomal abnormality in Philadelphia was discovered in 1959, it took decades to combine the science and targeted treatment into a marketable and successful agent. The author depicts the importance of the academics and Big Pharma working together as a team to test and accept the first truly targeted therapy for CML.

**Figure 4:**
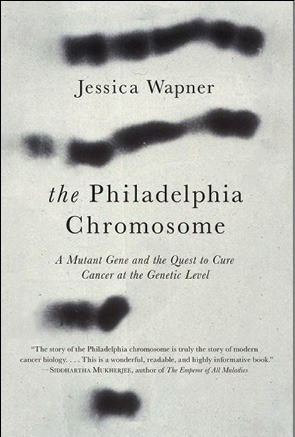
The Philadelphia Chromosome: A Mutant Gene and the Quest to Cure Cancer at the Genetic Level

## Autobiography of a Face

**Lucy Grealy** 

At age 9, Lucy Grealy was diagnosed with Ewing’s sarcoma. Although she made a full recovery after more than 2 years of grueling chemotherapy and radiation, she was left with only two-thirds of her jaw and a permanent facial disfigurement. Multiple surgeries over the next 20 years had only limited success, and Grealy struggled for the rest of her life to overcome the mental scars that her facial anomaly had brought about: "I spent 5 years of my life being treated for cancer, but since then I’ve spent 15 years being treated for nothing other than looking different from everyone else. It was the pain from that, from feeling ugly, that I always viewed as the great tragedy of my life. The fact that I had cancer seemed minor in comparison." Grealy presents us with a story that is not easy to forget. 

**Figure 5:**
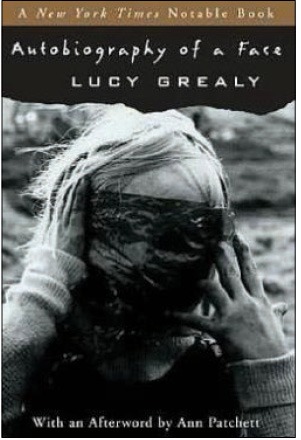
Autobiography of a Face

## Toms River: A Story of Science and Salvation

**Dan Fagin** 

Toms River is a fascinating look at toxic industrial plants and the greed of corporations who manufactured chemicals in this quiet New Jersey coastal community. Veteran environmental journalist Dan Fagin describes his meticulous research into cancer hot spots and their effects on local children, and the massive amount of contamination that occurred in the region following the arrival of these chemical plants in the early 1950s. This compelling book is a frightening examination of the cancer epidemiology of the Toms River families, their brave struggle to get the government to study and reveal the dangers of the region, and the subsequent settlement that occurred. Although you probably already know the outcome to this story, this gripping human drama is at the same time a very important book on industrial contamination and the dangers of exposure to toxic chemicals.

**Figure 6:**
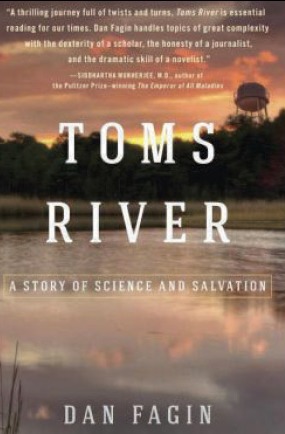
Toms River: A Story of Science and Salvation

## My Own Country: A Doctor’s Story

**Abraham Verghese** 

In a searing memoir, Abraham Verghese (author of the acclaimed novel Cutting for Stone) describes the experiences he had in the 1980s, working in the early days of the AIDS epidemic in Johnson City, Tennessee. Verghese recounts his patients’ stories and candidly weaves in his own feelings regarding AIDS, at-risk populations, and his own family’s fears about his health and possible exposure working with these patients. With great honesty and humility, Verghese elegantly traces his growth and personal perspectives about a then-unknown disease that stigmatized and separated patients from their friends, family, and fellow community members.

**Figure 7:**
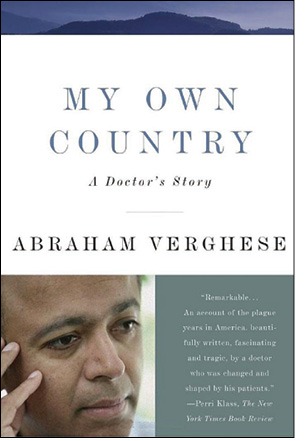
My Own Country: A Doctor’s Story

## The Checklist Manifesto: How to Get Things Right

**Atul Gawande** 

Atul Gawande is an extremely engaging writer. He manages to describe things in an accessible and entirely understandable way and makes such sense that one wonders why we haven’t adopted every suggestion he makes in this book. Some of the failures that occur in our lives every day can be prevented by the simplest of remedies: the checklist. And nowhere is the checklist more drastically needed than in health care! Gawande describes how checklists can improve a variety of situations and readers will note how easily they can be implemented in different settings, including our own health-care facilities. This is a very helpful read!

**Figure 8:**
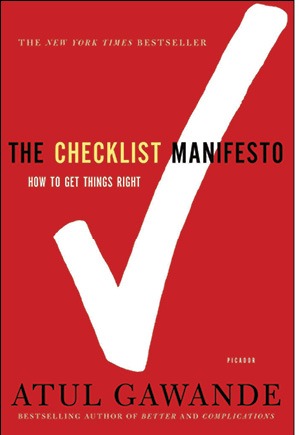
The Checklist Manifesto: How to Get Things Right

